# Embryonic Stem Cell Differentiation to Functional Arterial Endothelial Cells through Sequential Activation of ETV2 and NOTCH1 Signaling by HIF1α

**DOI:** 10.1016/j.stemcr.2017.07.001

**Published:** 2017-08-03

**Authors:** Kit Man Tsang, James S. Hyun, Kwong Tai Cheng, Micaela Vargas, Dolly Mehta, Masuko Ushio-Fukai, Li Zou, Kostandin V. Pajcini, Jalees Rehman, Asrar B. Malik

**Affiliations:** 1 Department of Pharmacology and the Center for Lung and Vascular Biology, University of Illinois College of Medicine, 835 S. Wolcott, Chicago, IL 60612, USA

**Keywords:** vascular biology, endothelium/vascular type, stem cells, angiogenesis, developmental biology, cell therapy, hypoxia, Notch, differentiation

## Abstract

The generation of functional arterial endothelial cells (aECs) from embryonic stem cells (ESCs) holds great promise for vascular tissue engineering. However, the mechanisms underlying their generation and the potential of aECs in revascularizing ischemic tissue are not fully understood. Here, we observed that hypoxia exposure of mouse ESCs induced an initial phase of HIF1α-mediated upregulation of the transcription factor *Etv2*, which in turn induced the commitment to the EC fate. However, sustained activation of HIF1α in these EC progenitors thereafter induced NOTCH1 signaling that promoted the transition to aEC fate. We observed that transplantation of aECs mediated arteriogenesis in the mouse hindlimb ischemia model. Furthermore, transplantation of aECs in mice showed engraftment in ischemic myocardium and restored cardiac function in contrast to ECs derived under normoxia. Thus, HIF1α activation of *Etv2* in ESCs followed by NOTCH1 signaling is required for the generation aECs that are capable of arteriogenesis and revascularization of ischemic tissue.

## Highlights

• Hypoxia enhances ESC to EC differentiation via HIF1α-ETV2 signaling
• HIF1α directs arterial endothelial specification by upregulating Notch signaling
• Transplantation of hypoxia-derived aECs induces arteriogenesis in hindlimb ischemia
• Hypoxia-derived aECs restore cardiac function following myocardial infarction

## Graphical Abstract

**Figure:**
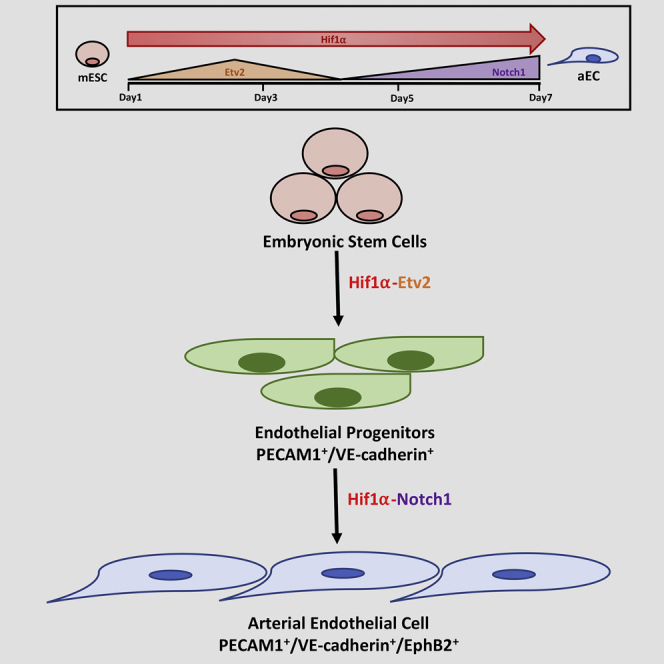

## Teaser

Tsang, Hyun, and colleagues demonstrate that hypoxic activation of the transcription factor HIF1α regulates endothelial differentiation via the vascular development transcription factor *Etv2*. Continued HIF1α signaling instructs arterial specification of endothelial cells via the Notch pathway. Transplantation of hypoxia-derived arterial endothelial cells promotes arteriogenesis and restores heart function following ischemic injury, demonstrating the essential role of hypoxia in arterial endothelial differentiation.

## Introduction

Stabilization of the transcription factor hypoxia-inducible factor 1α (HIF1α) in response to hypoxia induces expression of downstream targets regulating vasculogenesis, angiogenesis, and arteriogenesis (Semenza and Wang, 1992) (Bosch-Marce et al., 2007, Wang and Semenza, 1995). *Hif1α*^*−*/*−*^ mice are embryonically lethal whereas *Hif1α* heterozygotes (*Hif1α*^*+*/−^), although viable, are defective in expressing the angiogenic factor vascular endothelial growth factor (VEGF) and fail to induce adaptive arteriogenesis in the mouse hindlimb ischemia model (Bosch-Marce et al., 2007). Hypoxia participates in the EC fate commitment of ESCs (Kusuma et al., 2014, Prado-Lopez et al., 2010). Studies showed that hypoxia-primed embryoid bodies more efficiently differentiated into ECs than normoxic bodies (Lee et al., 2012). Multiple mechanisms are thought to be involved in the generation of ECs. Hypoxia increases the production of VEGF, basic fibroblast growth factor (bFGF), angiopoietin-1, and platelet-derived growth factor, all of which are linked to EC generation (Shin et al., 2011). Another mechanism of EC generation involves the induction of the Notch ligand *Dll4* and Notch target genes *Hey1* and *Hey2* (Diez et al., 2007). Studies in zebrafish and mice showed that activation of Notch signaling was critical for specifying arterial EC (aEC) fate differentiation during embryogenesis (Lawson et al., 2001, Lawson et al., 2002). Ets transcriptional factor family members can also regulate vasculogenesis and angiogenesis (Oikawa and Yamada, 2003, De Val and Black, 2010) (Abedin et al., 2014) (Shi et al., 2014), indicative of their role in specifying EC fate. Deletion of the Ets family member *Etv2* in particular impaired vasculogenesis in mice (Lee et al., 2009). Studies showed that *Etv2* activation functioned through the expression of *Dll4*, and thus contributed to aEC differentiation (Wythe et al., 2013).

While HIF-1α and ETV2 signaling induce differentiation of ESCs to ECs, the mechanisms specifying the differentiation to aECs and whether aEC generation requires sequential activation of a specific set of signaling pathways have not been addressed. Here, we demonstrate that hypoxia-mediated EC differentiation from ESCs occurs in a biphasic manner, initially inducing HIF1α-dependent *Etv2* activation that promotes ESC commitment to the EC lineage and then directing EC progenitors to the aEC fate via activation of NOTCH1 signaling. Upon transplantation, aECs were capable of inducing arteriogenesis in the mouse hindlimb ischemia model as well as engrafting and restoring the function of ischemic myocardium in mice. Thus, activation of the HIF1α-ETV2-NOTCH1 signaling axis in ESCs is an essential mechanism of differentiation to aECs that are capable restoring arterial perfusion and function of ischemic tissue.

## Results

### Hypoxia Enhances Endothelial Cell Generation from ESCs

**Figure 1:**
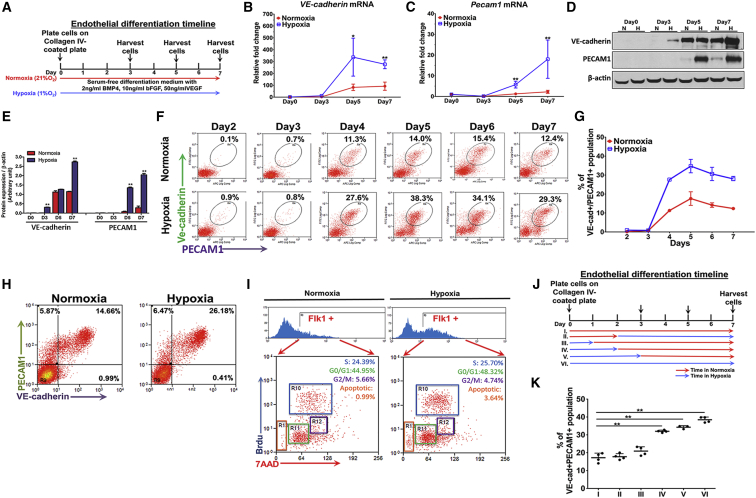
Hypoxia Induces ESC Differentiation to Endothelial Cell Fate (A) Schematic showing the timeline of EC differentiation protocol from mESCs. (B and C) mRNA expression of *VE-cadherin* and *Pecam1* detected by real-time PCR on days 0, 3, 5, and 7 during differentiation (n = 3 independent experiments). (D and E) Representative immunoblot and protein quantification of VE-cadherin and PECAM1 on days 0, 3, 5, and 7 of endothelial differentiation (n = 3 independent experiments). (F and G) Increased percentage of VE-cadherin^+^/PECAM1^+^ population was detected by flow cytometry at day 4 to day 7 under hypoxia (1% O_2_) Data shown as percentage of VE-cadherin^+^/PECAM1^+^ population in normoxia and hypoxic differentiation (n = 3 independent experiments). (H) Enhanced VE-cadherin^+^/PECAM1^+^ population at day 8 of endothelial differentiation from hESCs under hypoxia stimulus (n = 3 independent experiments). (I) Cell-cycle analysis of FLK1^+^ endothelial progenitor cells derived during normoxia; hypoxia did not augment proliferation of differentiating cells (n = 3 independent experiments). (J and K) Diagram and statistical analysis demonstrating the six conditions used to test the importance of short and early hypoxic exposure in mediating of EC differentiation. Red arrows indicate the duration of normoxic period; Blue arrows indicate the duration of hypoxic period. Doubling of VE-cadherin^+^/PECAM1^+^ ECs as determined by flow cytometry at day 7 of differentiation after a 2-day period of hypoxia (condition IV) when compared with normoxia (condition I). Note that a 3-day period of hypoxic exposure (condition V) was as effective in generating ECs as a full 7 days of hypoxia (condition VI) (n = 4 independent experiments). Data are shown as means ± SD. ^∗^p ≤ 0.05, ^∗∗^p ≤ 0.01.

Mouse ESCs (mESCs) were differentiated to ECs using bone morphogenetic protein 4, bFGF, and VEGF in either 1% O_2_ or 21% O_2_ conditions (Figure 1A). ECs were identified by cell-surface expression of VE-cadherin and PECAM1. Both mRNA and protein levels of these markers were significantly greater in the hypoxic cells at day 5 and day 7 of differentiation than in ESCs differentiated under normoxia (Figures 1B–1E). We found by flow cytometry that hypoxia doubled the generation VE-cadherin^+^/PECAM1^+^ cells compared with normoxia cells (Figures 1F and 1G). The enhanced generation of ECs during hypoxic differentiation of mESCs was also seen using human ESCs (hESCs) (Figure 1H). We next performed a bromodeoxyuridine (BrdU) assay to determine whether the ECs generated under hypoxia were due to increased proliferation or differentiation. As shown in Figure 1I, hypoxia did not augment proliferation of differentiating cells, indicating that the increase in ESC-derived ECs could not be attributed to increased proliferation of ECs.

We also addressed whether duration of hypoxia exposure per se was a requirement in mediating EC differentiation (Figures 1J and 1K). Here we determined the effects of (I) continuous normoxia for 7 days, (II) 2 days of normoxia followed by 5 days of hypoxia, (III) 1 day of hypoxia followed by 6 days of normoxia, (IV) 2 days of hypoxia followed by 5 days of normoxia, (V) 3 days of hypoxia followed by 4 days of normoxia, and (VI) 7 days of continuous hypoxia. We observed that a minimum of 2 days of hypoxic exposure was required to increase EC differentiation from ESCs (Figure 1K). Importantly, the initiation of hypoxia after the 2-day period of normoxia failed to increase EC generation, indicating an early 2-day window during differentiation when hypoxia was critical for the augmentation of EC generation.

### HIF1α Signaling and Early-Onset *Etv2* Expression Mediates Endothelial Cell Commitment

**Figure 2:**
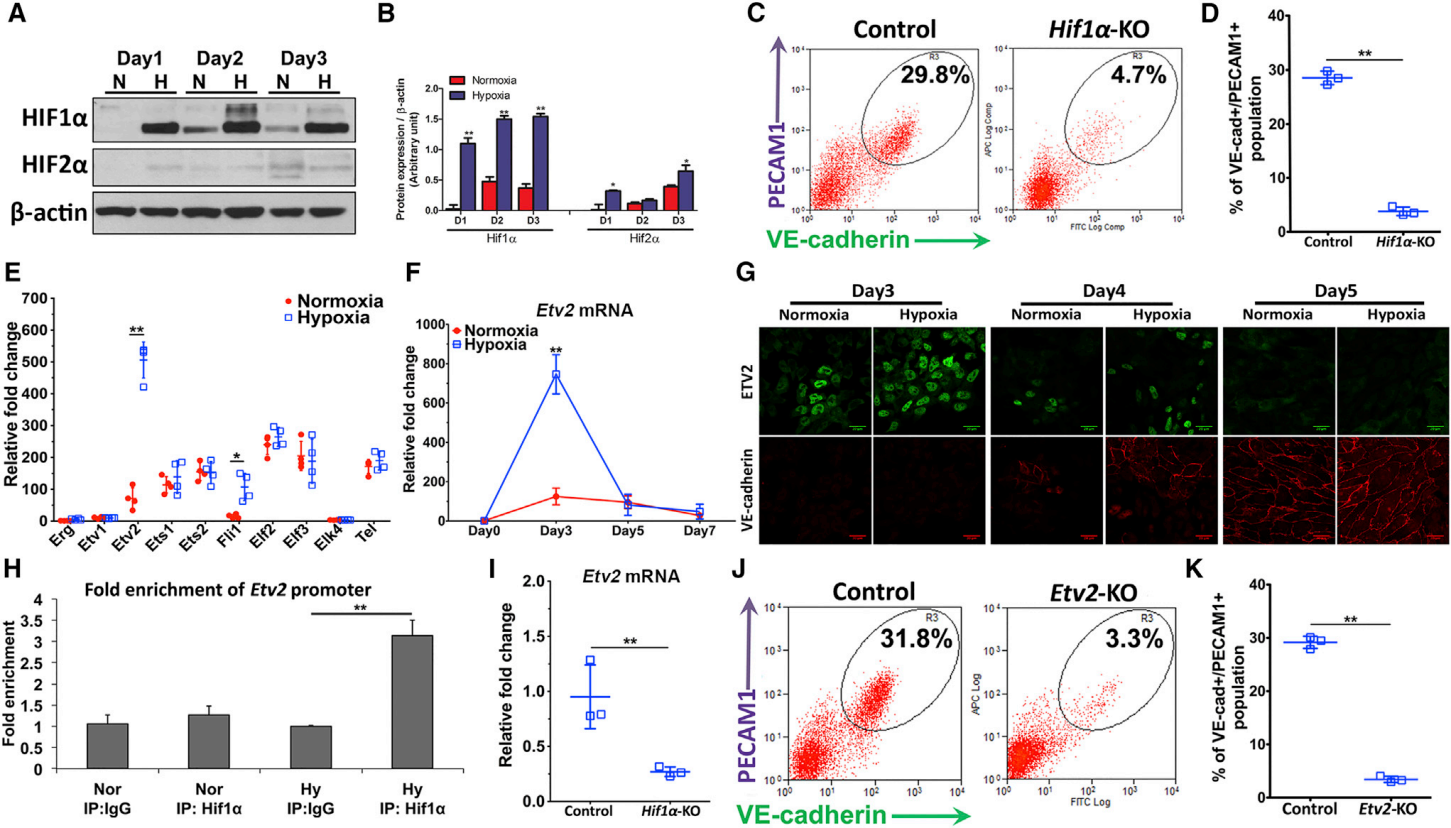
HIF1α Upregulates *Etv2* Expression and Augments Differentiation Endothelial Cell Progenitors (A and B) Protein expression of HIF1α and HIF2α on day 1 to day 3 of total population under differentiation. Early protein stabilization of HIF1α was sustained from day 1 to day 3 under hypoxia stimulation (n = 4 independent experiments). (C and D) *Hif1α*^*−*/*−*^ mESCs were obtained using CRISPR/Cas9 with *Hif1α*-specific gRNA. Percentage of VE-cadherin^+^/PECAM1^+^ population was significantly decreased in *Hif1α*^*−*/*−*^ mESCs compared with control mESCs (n = 3 independent experiments). (E) mRNA expression of 10 Ets family members were screened at day 3 of differentiation. Only *Etv2* and *Fli1* were significantly increased during hypoxia (n = 4 independent experiments). (F) Time course of changes in mRNA expression of *Etv2* demonstrated that hypoxia-induced early expression of *Etv2* as opposed to normoxia (n = 3 independent experiments). (G) Representative protein expression of ETV2 detected by immunofluorescence indicate ETV2 expression and nuclear localization at day 3, which declined by day 4 and disappeared by day 5 of differentiation (n = 3 independent experiments). (H) Enrichment of *Etv2* promoter using anti-HIF1α antibody in a chromatin immunoprecipitation (IP) assay shows binding of HIF1α to *Etv2* promoter (n = 3 independent experiments). IgG, immunoglobulin G. (I) Knockout of *Hif1α* reduced expression of *Etv2* at day 3 of hypoxic differentiation (n = 3 independent experiments). (J and K) *Etv2*^*−*/*−*^ mESCs were obtained using CRISPR/Cas9 with *Etv2*-specific gRNA (J). Percentage of VE-cadherin^+^/PECAM1^+^ population was significantly decreased in Etv2^*−*/*−*^ mESCs when compared with control. (K) Percentage of VE-cadherin^+^/PECAM1^+^ cells on day-7 hypoxic EC differentiation of *Etv2*^*−*/*−*^ mESCs (n = 3 independent experiments). Data are shown as means ± SD. ^∗^p ≤ 0.05, ^∗∗^p ≤ 0.01.

To test the role of HIF signaling in endothelial differentiation of mESCs, we first determined the expression levels of HIF1α and HIF2α in mESCs differentiated under hypoxic and normoxic conditions. We observed that HIF1α but not HIF2α was upregulated during the early phase of EC differentiation under hypoxia (Figures 2A and 2B). HIF1α activation was characterized by the upregulation of its target genes, which regulate glycolysis such as the glucose transporter (*GLUT1*), isoforms of pyruvate dehydrogenase kinase (*PDK1*, *3*, *4*) and lactate dehydrogenase (*LDH-A*) (Schödel et al., 2011, Semenza, 2003). We therefore assessed the expression of these metabolic genes and found their upregulation, consistent with increased HIF1α activation (Figure S1). Moreover, *Hif1α* deletion using CRISPR prevented the augmentation of differentiation into ECs (Figures 2C, 2D, and S2). We next addressed whether the early response was the result of HIF1α-mediated expression of Ets transcription factors known to regulate EC differentiation (Dejana et al., 2007, Marcelo et al., 2013, De Val and Black, 2010) We observed using a qPCR screen that the Ets factor *Etv2* was the most prominently upregulated isoform in ESCs undergoing differentiation during hypoxia (Figure 2E). *Etv2* expression under hypoxia was increased 7-fold at 2–3 days of hypoxia compared with normoxia (Figure 2F). Immunofluorescence also showed nuclear translocation of ETV2 in the hypoxia-exposed cells during this period (Figure 2G). The *Etv2* response during hypoxia was, however, short-lived as it sharply decreased at day 5 to the levels seen in normoxic cells (Figure 2F).

Using the chromatin immunoprecipitation assay, we determined the role of HIF1α in regulating *Etv2* expression. We observed the binding of HIF1α to the *Etv2* promoter during hypoxia (Figure 2H). Deletion of *Hif1α* prevented *Etv2* expression (Figure 2I). To determine whether *Etv2* acted downstream of *Hif1α*, we generated *Etv2*^*−*/*−*^ mESCs. Deletion of *Etv2* in mESCs during hypoxia reduced EC differentiation by 6-fold (Figures 2J and 2K) without a reduction in *Hif1α* expression (Figure S3). Thus, HIF1α binding to *Etv2* promoter and *Etv2* expression were required for hypoxia-induced enhancement of EC progenitor differentiation.

### HIF1α Signaling Directs Specification to Arterial Endothelial Cell Fate

**Figure 3:**
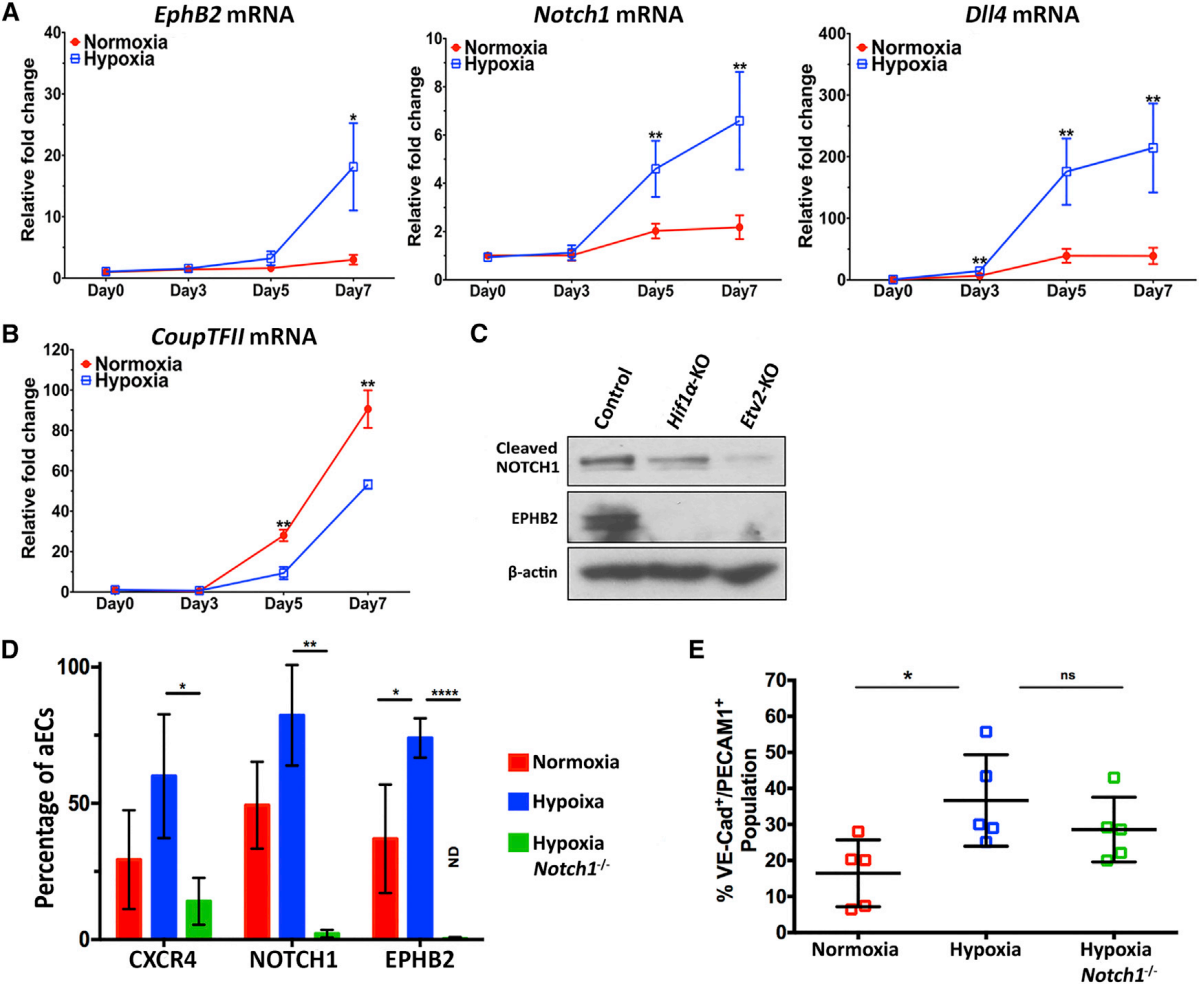
HIF1α in the Second Phase Directs Differentiation of ESCs to Arterial EC Fate via NOTCH1 Signaling (A and B) mRNA expression of arterial EC markers *EphrinB2*, *Notch1*, and *Dll4*, and the venous EC marker *Coup-TFII* on days 0, 3, 5, and 7 of EC differentiation (n = 3 independent experiments). Results show preferential generation of aECs during hypoxia as opposed to venous-type ECs. (C) Representative immunoblot of decreased expression of arterial EC markers EPHB2 and cleaved NOTCH1 in hypoxia-differentiated *Hif1α* ^*−*/*−*^ and *Etv2*^*−*/*−*^ mESCs (n = 4 independent experiments). (D) Percentage of arterial ECs in the endothelial differentiated pool at day 7. ECs were sorted by surface PECAM1 expression and analyzed for aEC markers CXCR4, NOTCH1, and EPHB2. Results show that hypoxic differentiation preferentially generates aECs and *Notch1* deletion ablates aEC formation (n = 3 independent experiments). (E) Percentage of VE-cadherin^+^/PECAM1^+^ endothelial progenitors differentiated from mESCs on day 7. Results show that deletion of *Notch1* in mESCs inhibit aEC formation but does not affect EC generation (n = 5 independent experiments). Data are shown as means ± SD. ^∗^p ≤ 0.05, ^∗∗^p ≤ 0.01, ^∗∗∗∗^p ≤ 0.0001; ns, not significant.

We next addressed maturation of EC progenitors under sustained hypoxia stimulus. Here we followed the aEC markers *Ephb2*, *Notch1*, and *Dll4* (Duarte et al., 2004, Gerety et al., 1999, Krebs et al., 2000, Shutter, 2000) and venous EC (vEC) marker *CoupTFII* (You et al., 2005). We observed by qPCR that the expression of aEC markers *EphrinB2*, *Notch1*, and *Dll4* (Park et al., 2013, Swift and Weinstein, 2009) increased between days 3 and 7 of hypoxia (Figure 3A). The increase in these markers was mirrored by decreased *Etv2* levels on day 5 (Figure 2F), consistent with a maturation of ECs during the continuing hypoxia. In contrast to aEC markers, expression of vEC marker *CoupTFII* was lower in hypoxic cells (Figure 3B). We also observed, importantly, that deletion of either *Hif1α* or *Etv2* prevented the generation of aECs (Figure 3C).

We next determined whether hypoxia enhanced aEC specification through NOTCH1 signaling. Here we observed that *Notch1*^*−*/*−*^ ESCs failed to augment aEC differentiation during hypoxia as evidenced by no increase in the aECs markers CXCR4, NOTCH1, and EPHB2 (Figure 3D). Importantly, *Notch1* deletion in mESCs did not suppress hypoxia-induced EC progenitor differentiation (Figure 3E), indicating that NOTCH1 only regulated aEC lineage commitment and not the initial phase of endothelial differentiation.

### aEC Transplantation Induces Arteriogenesis and Restores Cardiac Function Post Infarction

**Figure 4:**
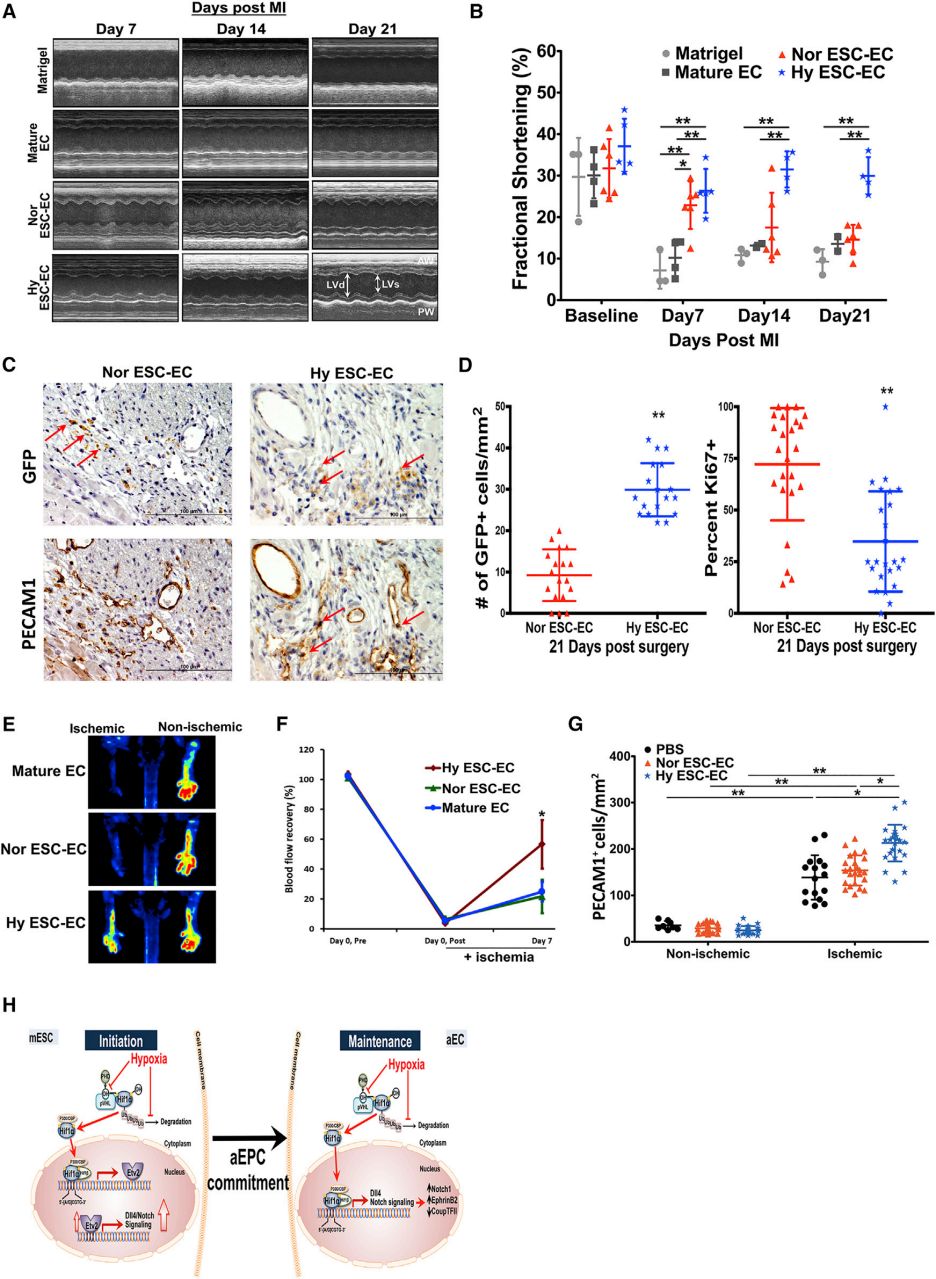
Hypoxia-Derived ECs Improve Perfusion of Ischemic Hindlimb via Arteriogenesis and Cardiac Function after Myocardial Infarction in Mice (A) Representative M-mode echocardiographic views of infarcted mouse hearts 1 week, 2 weeks, and 3 weeks after induction of myocardial infarction; n = 3 in Matrigel group. n = 4 in mature EC group. n = 6 in normoxia-derived ESC-EC (Nor ESC-EC) group, and n = 5 in hypoxia-derived ESC-EC (Hy ESC-EC) group. LVd, left ventricular diastolic dimension; LVs, left ventricular systolic dimension. (B) Quantification of fractional shortening (%) calculated as ([LVd −LVs]/LVd × 100) in mice tested in (A). Mice injected with Hy ESC-EC showed sustained improvement of cardiac function (^∗^p < 0.05, ^∗∗^p < 0.01); results are shown as mean ± SD. (C) Representative images (40×) of serial heart sections (0.5 μm apart) stained by immunochemistry for GFP^+^ ECs and PECAM1 to identify host vasculature. Results indicate more hypoxia ESC-ECs than normoxia ESC-ECs engraftment within PECAM1-stained vessels (red arrows). (D) Quantification and proliferation analysis of transplanted normoxia ESC-ECs and hypoxia ESC-ECs on 21-day infarcted hearts. Data were obtained from three animals and with at least six fields examined from each animal; results are shown as mean ± SD (^∗∗^p < 0.01). (E) Serial laser Doppler perfusion imaging analysis in ischemic and non-ischemic limbs of mice (n = 3 mice per group). (F) Quantification of hindlimb blood flow recovery determined by calculating ischemic/non-ischemic perfusion ratios before and after surgery. Beneficial effects of injected hypoxia ESC-ECs in ischemic-induced injury are clearly evident (^∗^p < 0.05); results are shown as mean ± SD. (G) Quantification of number of PECAM1^+^ cells in gastrocnemius (GC) muscles of non-ischemic and ischemic hind limbs. Injection of hypoxia ESC-EC enhanced vessel regeneration after ischemia when compared with normoxia ESC-ECs (^∗^p < 0.05, ^∗∗^p < 0.01). Data were obtained from three mice and eight fields from each animal; results are shown as mean ± SD. (H) Model of generation of aECs under hypoxia. Under hypoxia, the stabilization of HIF1α in mESCs in the initial phase leads to *de novo* expression of *Etv2* by binding to hypoxia response elements (HRE) within the *Etv2* promoter. *Etv2* expression augments EC commitment. Persistent stabilization of HIF1α under hypoxia subsequently maintained the hypoxia response. HIF1α is recruited to the HRE in promoter of *Dll4* and activates NOTCH1, resulting in commitment to arterial EC subtype via upregulation of EPHB2 as well as suppression of *CoupTFII*, which is restricted to venous-type ECs.

To determine whether hypoxia-derived aECs could induce arteriogenesis and were efficacious compared with normoxia-derived ECs when transplanted in ischemic tissue, we utilized the mouse hindlimb ischemia model (Urao et al., 2008) and a mouse myocardial infraction model (Mavrommatis et al., 2013). To track the injected cells, we generated an mESC reporter line using CRISPR/Cas9 to incorporate the GFP gene within exon 1 of *Pecam1* gene; GFP expression in these cells therefore marked the generation of ECs following the 7-day differentiation conditions and allowed us to track the fate of the cells following cell transplantation *in vivo*. We observed by flow cytometry that 90% of the GFP^+^ cells co-stained with anti-PECAM1 and anti-VE-cadherin antibodies (Figure S4), thus validating the EC reporter. ECs (1 × 10^6^) generated under normoxia and hypoxia were transplanted into ischemic hindlimbs following femoral artery ligation. We observed markedly improved blood flow recovery on day 7 in the hindlimbs receiving aECs compared with normoxia-derived ECs (Figure 4F). Further analysis of the gastrocnemius muscle on day 28 revealed an increased number of PECAM1^+^ blood vessels in ischemic tissue transplanted with aECs, helping to explain the increased perfusion seen on day 7 (Figure 4G). We also compared the differential therapeutic efficacy of ECs generated in normoxic and hypoxic condition on cardiac function following myocardial infarction by implanting 5 × 10^5^ cells into infarcted mouse hearts. Echocardiography of mice transplanted with normoxia-derived GFP^+^ ECs on week1 showed modest improvement (Figures 4A and 4B). However, this improvement was transient as it was fully reversed by week 2 (Figure 4B). In sharp contrast, hypoxia-derived GFP^+^ ECs significantly increased and sustained the improvement in cardiac function over 21-day study period (Figures 4A and 4B). Immunohistochemistry performed on serial sectioned hearts slides 21 days after surgery was used to visualize and quantitate the transplanted GFP^+^ cells in relation to the surrounding vasculature. Although an identical number of normoxic and hypoxic differentiated ECs were transplanted, a greater number of hypoxia-derived GFP^+^ ECs persisted and showed increased engraftment in the host endothelium throughout the 21-day study period (Figures 4C and 4D). Further analysis of GFP^+^ ECs in day 21 heart sections with the proliferation marker Ki-67 showed lower levels of proliferation in hypoxia-derived ECs (Figure 4D), suggesting that the increased engraftment and revascularization by hypoxia-derived ECs was due to their enhanced functional capacity and not to increased proliferation.

## Discussion

In this study, we demonstrate the biphasic and sequential role of HIF1α signaling. HIF1α first generates EC progenitors by upregulating the transcription factor *Etv2* and subsequently drives maturation to an aEC fate via a NOTCH1-dependent signaling mechanism. We also show that the hypoxia-induced augmentation of EC generation was due to enhanced differentiation and arterial fate commitment, as opposed to increased proliferation. Transplantation of aECs differentiated under hypoxia induced arteriogenesis in the mouse hindlimb ischemia model and engraftment of aECs coupled with improved function of infarcted mouse hearts.

The current model of arterial and venous specifications is VEGF-centric, whereby angioblasts exposed to increasing concentrations of VEGF adopt an aEC fate (Fish and Wythe, 2015). In the present study, we demonstrated that mESCs grown in hypoxia had a greater propensity to differentiate to aECs in the presence of high concentrations of VEGF than mESCs differentiated under normoxia. HIF1α activation of *Etv2* and subsequently of NOTCH1 signaling was required for aEC generation. Interestingly, ESCs were most sensitive to hypoxia in the first 2 days of differentiation, suggesting the importance of an early hypoxia window in epigenetically imprinting ESCs for their subsequent aEC fate. Hypoxia during this phase may modify the epigenetic landscape through the expression of histone demethylases KDM4A and KDM4C, which were shown to be important for transitioning ESCs to ECs (Wu et al., 2015). Our observation that 2 days of hypoxia was required to specify the aEC fate is consistent with the expression of *KDM4A* and *KDM4C*, which peaked on days 2 and 5 during EC differentiation (Wu et al., 2015); that is, expression of these histone demethylases occurred before the expression of *Etv2* in the present study. We also found that the hypoxia-differentiated ECs exhibited upregulation of the glycolytic genes, glucose transporter 1 (*Glut1*) and lactate dehydrogenase (*Ldha*). This may also contribute to subsequent endothelial differentiation, since ECs primarily rely on glycolysis to meet their ATP demands (Eelen et al., 2016).

Our results show that generation of aECs follows a distinct sequential process requiring initial HIF1α-induced expression of *Etv2* to transition ESCs to EC progenitors followed by activation of NOTCH1 signaling that induces the generation of aECs. The decrease in *Etv2* expression interestingly preceded HIF1α-induced expression of NOTCH1. The role of *Notch1* in generating aECs is consistent with NOTCH1's fundamental importance in arterial differentiation during embryonic development (Lawson et al., 2001). The changes in the expression of *Etv2* and subsequently in *Notch1* expression recapitulated those observed during embryonic arteriogenesis (Wythe et al., 2013); thus, hypoxia-induced ESC to aEC transition recapitulated the essential elements of arterial differentiation during embryonic vascular development.

*Etv2* is a developmental transcription factor mediating EC progenitor formation between embryonic days 7.5 and 9.5 in mice (Lee et al., 2009) (Ferdous et al., 2009). Upregulation of *Etv2* expression was required for the initiation of embryonic vasculogenesis; however, *Etv2* is subsequently downregulated prior to vessel maturation (Ferdous et al., 2009, Lee et al., 2009). We observed moderate upregulation and subsequent downregulation of *Etv2* during differentiation of ESCs into ECs under normoxia: however, the amplitude of temporal *Etv2* expression was markedly enhanced under hypoxic conditions and mirrored the *in vivo Etv2* expression pattern seen during embryonic vasculogenesis (Ferdous et al., 2009) (Kataoka et al., 2011)^.^ Hypoxia-induced upregulation of *Etv2* expression also resulted in greater Etv2 transcriptional activity as evidenced by hypoxia-induced increases in the expression of known ETV2 targets *Flk1*, *Pecam1*, and *VE-cadherin* (Kohler et al., 2013, Lee et al., 2009, De Val and Black, 2010). We observed that genetic deletion of *Etv2* in ESCs abrogated the hypoxia-induced EC generation, supporting the requisite role of *Etv2* during ESC to EC differentiation. Furthermore, deletion of *Hif1α* in ESCs prevented both *Etv2* upregulation as well as EC differentiation. These data together demonstrate that HIF1α-induced *Etv2* expression during the early phase of hypoxia is the primary determinant of mESC differentiation to EC progenitors.

Hypoxia acted in a biphasic manner during the endothelial differentiation program, first enhancing HIF1α-ETV2-dependent generation of EC progenitors and then instructing these progenitors toward the aEC fate. As in the initial phase EC progenitor differentiation, the generation of aECs was also HIF1α dependent. Stabilization of HIF1α in the committed EC progenitors activated expression of *Notch1* and Notch ligand *Dll4*, known to be required for arterial differentiation during development (Lawson et al., 2001). We observed that deletion of *Hif1α* significantly reduced EPHB2 expression, an aEC marker, indicating a requirement for persistent HIF1α activity in the aEC specification mechanism. That HIF1α signaling induced generation of EC progenitors via *Etv2* and subsequently aECs via *Notch1* is in agreement with studies showing that NOTCH1 signaling mediates arterial fate specification during development (Park et al., 2013, Shutter, 2000, Swift and Weinstein, 2009, You et al., 2005). Studies have also shown that vasculogenesis is initiated in *Dll4*^*−*/*−*^ (Duarte et al., 2004), *Notch1*^*−*/*−*^ (Krebs et al., 2000, Swiatek et al., 1994), and *Hey1*^*−*/*−*^/*Hey2*^*−*/*−*^ mice (Fischer et al., 2004), but arterial blood vessels failed to form, consistent with the observed essential role of Notch signaling in aEC fate determination.

We observed that the levels of *EphB2*, *Notch1*, and *Dll4* remained elevated for the 7-day hypoxia period, whereas expression of the venous marker *CoupTFII* was reduced during this period to the levels seen during normoxia. A possible explanation for this finding is that hypoxia-mediated upregulation of *Dll4* and *Hey2* leads to repression of *CoupTFII* (Diez et al., 2007). Thus, sustained hypoxia in the present study induced activation of the DLL4-NOTCH-HEY2 signaling pathway, which in turn may repress *CoupTFII* and thereby favor the generation of aECs.

Because aECs generated during hypoxia may be useful in revascularizing ischemic tissue, we assessed their capacity in two different ischemia models. We ensured that the same number of normoxia- or hypoxia-derived ECs was transplanted. In the mouse myocardial infarction model, transplantation of hypoxia-derived GFP^+^ ECs resulted in long-term persistence, indicating their engraftment in ischemic tissue and improved cardiac function following myocardial infarction. Furthermore, in the mouse hindlimb ischemia model, transplantation of hypoxia-derived ECs induced arteriogenesis and formed neovessels throughout the 28-day study period when compared with normoxia-derived ECs. As transport of oxygenated blood is an intrinsic behavior of arteries, the hypoxia-derived aECs appeared to be superior to the normoxia-derived cells in normalizing arterial perfusion. Ki-67^+^ analysis of GFP^+^ ECs in infarcted hearts 21 days after infarction showed hypoxia-derived ECs to be significantly less proliferative; thus, we attribute the increased perfusion and revascularization to the engraftment of aECs as opposed to proliferation of these cells per se. We cannot rule out the possibility that hypoxia-derived ESC-ECs may have demonstrated increased proliferation early on after the implantation which we could not detect; however, the lack of hypoxia-induced proliferation during the ESC-to-EC differentiation period prior to transplantation suggests that hypoxia does not augment ESC-EC proliferation. Another possibility for the long-term sequestration and functional improvement of aECs could be the enhanced survivability of the cells. As ischemic tissue presents a hypoxic environment and we showed (Figure S1) that cells undergoing hypoxic differentiation demonstrated significant increases in the expression of glycolytic genes, aECs may better tolerate the hypoxic environment of the ischemic tissue and thus be resilient to oxygen deprivation. A further indication that the hypoxia-derived aECs were indeed more angiogenic is evidenced by an increased number of new PECAM1^+^ vessels formed in the gastrocnemius in mouse hindlimb after transplantation of aECs.

A limitation of our study is that the hypoxic differentiation not only generated the aEC population but also activated hypoxic pathways, which could itself enhance the therapeutic efficacy of the transplanted cells independent of their arterial fate. Thus, it is possible that release of paracrine factors also contributes to the improved perfusion. It has been previously shown that CD34^+^/VE-cadherin^+^ cells derived from induced pluripotent stem cells release pro-angiogenic and anti-apoptotic factors (Gu et al., 2012), which may contribute to the recovery of cardiac function following myocardial infarction seen in the present study.

In conclusion, our results show the importance of hypoxic signaling in directing the sequential generation of EC progenitors to aECs. The first phase requires activation of HIF1α to mediate the expression of *Etv2*, which specifies EC progenitor fate. Sustained HIF1α activation also mediates the second phase resulting in expression of *Notch1*, which induces maturation of EC progenitors into aECs. These findings show a fundamental role for hypoxia signaling in aEC fate determination and points to an innovative approach for enhancing therapeutic efficacy of ESC-derived aECs in revascularizing ischemic tissue.

## Experimental Procedures

### mESC Culture and Endothelial Differentiation Protocol

J1 mESCs were cultured on a feeder layer of mouse embryonic fibroblasts. Prior to endothelial differentiation, cells were passaged onto 0.1% gelatin coated plates using the same medium. Undifferentiated H1 hESCs were cultured on Matrigel-coated plate with Essential 8 medium (Gibco). mESCs were seeded onto collagen IV-coated plates and maintained in endothelial cell differentiation medium for 7 days either under normoxia (21% O_2_) or hypoxia (1% O_2_). This differentiation protocol generates functional endothelial cells expressing endothelial transcription factors and endothelial-specific cell-surface markers such VE-cadherin, forming tubes *in vitro* and blood vessels *in vivo* (Toya et al., 2015). hESCs were seeded onto Matrigel-coated plates in Essential 8 medium and incubated for 24 hr (day 1). On the next day (day 0), Essential 8 medium was replaced with human endothelial cell differentiation medium and incubated for 3 days. At days 3, 5, and 7, the medium was replaced with serum-free differentiation medium supplemented with VEGF and SB431542 (Toya et al., 2015). Differentiated cells were collected on day 8 for analysis by flow cytometry.

### CRISPR/Cas9 Gene Deletion

mESCs were co-transfected with plasmids containing Cas9, *Hif1α*-specific guide RNA (gRNA) and GFP as well as homology-directed repaired plasmids containing homology arms corresponding to the cut sites generated by *Hif1α*-specific gRNA. GFP^+^ mESCs were sorted and cultured under puromycin selection. HIF1α protein expression of each clone was then assessed in hypoxia-exposed mESCs. An *Etv2*^*−*/*−*^ mESC line was generated by transfecting px330 plasmid (Cong et al., 2013) containing gRNA targeting *Etv2*. Resultant single mESC colonies were then picked and expanded. Each clone was subjected to EC differentiation protocol and *Etv2* expression in each clone was determined on day 3 to validate gene knockout. *Notch1*^*−*/*−*^ mESCs were also generated by co-transfecting px330 plasmid (Cong et al., 2013) containing *Notch1*-specific gRNA with a puromycin-containing plasmid. Individual colonies were expanded and *Notch1* deletion was validated by qPCR and western blot.

### *Pecam1*-GFP mESC Reporter Line

To track the fate of the injected cells *in vivo*, we generated a mESC reporter line using CRISPR/Cas9. GFP was incorporated within the exon 1 of *Pecam1* gene; GFP expression was driven by endogenous *Pecam1* promoter. The sequence of gRNA used for targeting mouse *Pecam1* locus was 5′-CAG CTG AGG TGG GCC TCA GT-3′.

### Cell-Cycle Analysis

BrdU labeling and flow cytometric quantification were used to determine proliferation of mESCs undergoing endothelial differentiation. FLK1 was used to gate for endothelial cells (Yamashita et al., 2000).

### Mouse Hindlimb and Cardiac Ischemia Models

The animal experiments were carried out according to approved protocols by the Animal Care Committee (ACC) at the University of Illinois, Chicago. *Pecam1*-driven GFP reporter mESC line was used to study the uptake of endothelial cells. At day 7 of endothelial differentiation, GFP^+^ cells were sorted by fluorescence-activated cell sorting. For the mouse myocardial infarction model, C57/BL6 mice were subjected to left main coronary artery ligation to induce infarction as described by Mavrommatis et al. (2013). Immediately after ligation, hypoxia-derived ECs, normoxia-derived ECs, primary mouse adult ECs (negative control), or Matrigel was introduced by direct intramuscular injections into the peri-infarcted regions of the myocardium. For the mouse hindlimb ischemia model, male athymic nude mice were subjected to unilateral hindlimb surgery as previously described (Urao et al., 2008). ESC-derived ECs or mature ECs were injected into three different spots in gastrocnemius muscles immediately after ischemic surgery. Ischemic (left)/non-ischemic (right) limb blood flow was measured using a laser Doppler blood flow analyzer as described by Urao et al. (2008).

### Statistical Analysis

All statistical analyses were performed with GraphPad Prism (GraphPad, San Diego). Results are expressed as mean ± SD. Differences between groups were analyzed using ANOVA and Student’s t test. p Values of less than 0.05 were considered statistically significant.

Full details of experiments are provided in Supplemental Experimental Procedures.

## Author Contributions

K.M.T., D.M., J.R., and A.B.M. designed the experiments; K.M.T., J.S.H., K.T.C., M.V., and L.Z. performed the experiments; K.M.T., J.S.H., K.T.C., L.Z., K.V.P., and M.U.-F. analyzed data; K.M.T., J.S.H., J.R., and A.B.M. wrote the paper. All authors reviewed the manuscript and made revisions or edits.

## Supplemental Information

Document S1. Supplemental Experimental Procedures, Figures S1–S4, and Table S1

Document S2. Article plus Supplemental Information
