# Supplementary material for: Embryonic Stem Cell Differentiation to Functional Arterial Endothelial Cells through Sequential Activation of ETV2 and NOTCH1 Signaling by HIF1α
Source: Stem Cell Reports. 2017 Aug 3;9(3):796–806. doi: 10.1016/j.stemcr.2017.07.001 (PMC5599266; doi:10.1016/j.stemcr.2017.07.001)
Supplement: Document S1. Supplemental Experimental Procedures, Figures S1–S4, and Table S1 [file mmc1.pdf]

Stem Cell Reports, Volume *9*

Supplemental Information

Embryonic Stem Cell Differentiation to Functional Arterial Endothelial Cells through Sequential Activation of ETV2 and NOTCH1 Signaling by HIF1α

Kit Man Tsang, James S. Hyun, Kwong Tai Cheng, Micaela Vargas, Dolly Mehta, Masuko Ushio-Fukai, Li Zou, Kostandin V. Pajcini, Jalees Rehman, and Asrar B. Malik

SUPPLEMENTAL INFORMATION

Figure S1

Analysis of glycolytic genes during hypoxic differentiation of mESC, Related to Figure 2

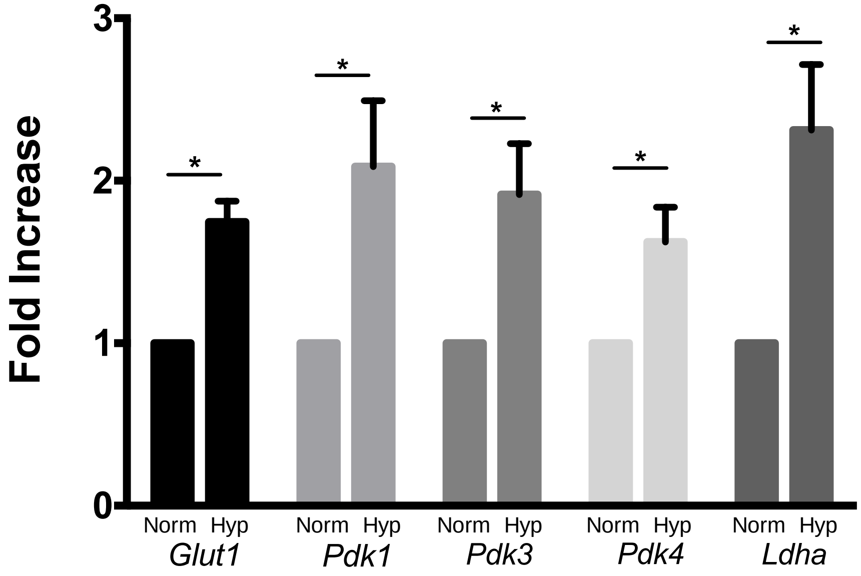

Figure S1 Related to Figure 2
qPCR analysis of Day7 cells undergoing normoxic and hypoxic endothelial cell differentiation. Expression of key glycolytic genes (Glucose Transporter1, Pyruvate Dehydrogenase1, Pyruvate Dehydrogenase3, Pyruvate Dehydrogenase4, Lactate Dehydrogenase A) were increased in hypoxic differentiation compared to normoxic differentiation. Results are representative of 3 independent biological replicates

Figure S2.

Levels of VE-cadherin during hypoxic differentiation of J1 and *Hif1a$^{-/-}$* mESCs, Related to Figure 2

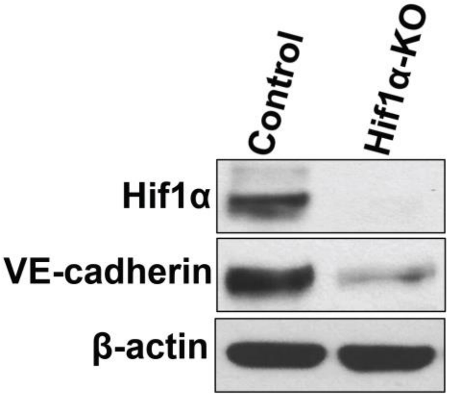

Figure S2 Related to Figure 2
HIF1α deletion of mouse embryonic stem cells reduces VE-cadherin expression at Day7 of hypoxia mediated endothelial differentiation. Results are representative of 3 independent experiments.

Figure S3.

Levels of HIF1α during hypoxic differentiation of J1, *Hif1α*$^{-/-}$, and *Etv2*$^{-/-}$ mESCs, Related to Figure 2

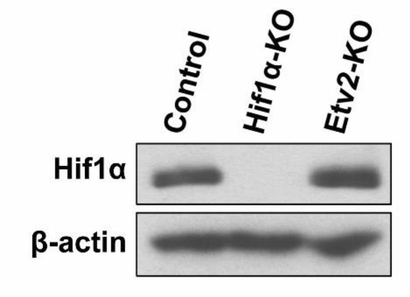

Figure S3 Related to Figure 2
Loss of *Etv2* in differentiating mESCs did not affect the expression of HIF1α, demonstrating *Etv2* acted downstream of *Hif1α*. Results are representative of 3 independent experiments.

Figure S4.

Validation of *Pecam1* mESC reporter, Related to Figure 4

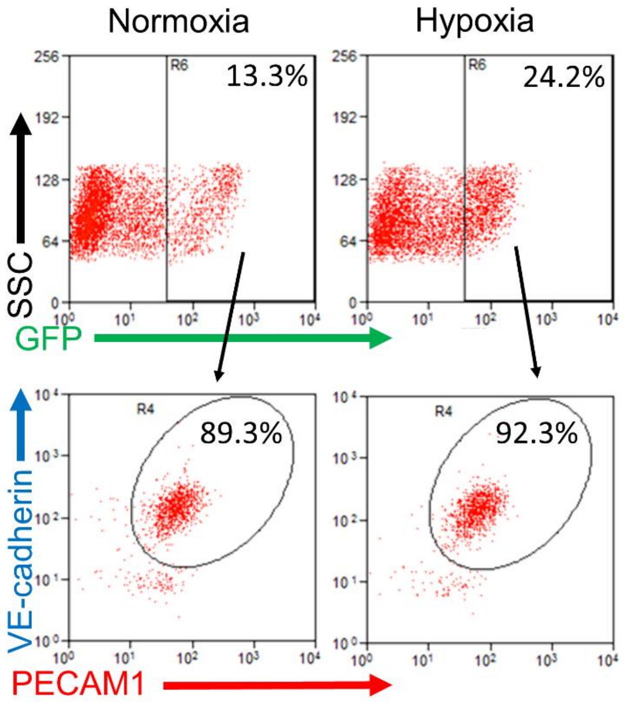

Figure S4 Related to Figure 4

Pecam1-GFP mESCs reporter were differentiated for 7days under normoxia and hypoxia then stained with PECAM1-APC and VE-cadherin-PE antibodies. ~90% GFP+ cells were positive for both PECAM1 and VE-cadherin, demonstrating that GFP+ cells were endothelial cells

Table S1.

List of primers used for mRNA quantification in real-time PCR, Related to Figure 1, 2, and 3

Gene	Primer Sequence 5'-3'
VE-cadherin	Forward: GGCCTAAGTGTGTCCTTGATTC<br>Reverse: TGGGTGAGAAGTATGGTGACTG
Pecam1	Forward: GAGCCCATTCACGTTTCAGTTT<br>Reverse: TCCTTCCTGCTTCTTGCTAGCT
Erg	Forward: ACCTCACCCCTCAGTCCAAA<br>Reverse: TGGTCGGTCCCAGGATCTG
Etv1	Forward: TTAAGTGCAGGCGTCTTCTTC<br>Reverse: GGAGGCCATGAAAAGCCAAA
Etv2	Forward: CAGAGTCCAGCATTCACCAC<br>Reverse: AGGAATTGCCACAGCTGAAT
Ets1	Forward: TCCTATCAGCTCGGAAGAACTC<br>Reverse: TCTTGCTTGATGGCAAAGTAGTC
Ets2	Forward: CCTGTCGCCAACAGTTTTCG<br>Reverse: TGGAGTGTCTGATCTTCACTGA
Fli1	Forward: ATGGACGGGACTATTAAGGAGG<br>Reverse: GAAGCAGTCATATCTGCCTTGG
Elf2	Forward: GTTCACAGCAGTAATGCTCACT<br>Reverse: TCAAGCAGGTAGGAGATTCCAT
Elf3	Forward: GCTGCCACCTGTGAGATCAG<br>Reverse: GTGCCAAAGGTAGTCGGAGG
Elk4	Forward: ATCTAACAATGGGGAGTTCAAGC<br>Reverse: GGCTCGGCTGAGTTTATCATAAT
Tel	Forward: AGCAGGAACGAATTTCATACACG<br>Reverse: GGCAGGTGGATCGAGTCTTC
EphrinB2	Forward: ATTATTTGCCCCAAAGTGGACTC<br>Reverse: GCAGCGGGGTATTCTCCTTC
Notch1	Forward: CTCCGTTACATGCAGCAGTT<br>Reverse: CCAGGATCAGTGGAGTTGTG
Dll4	Forward: TTCCAGGCAACCTTCTCCGA<br>Reverse: ACTGCCGCTATTCTTGTCCC
CoupTFII	Forward: ACCGGGTGGTCGCTTTTATG<br>Reverse: GGCCTTGAGGCAGCTATACTC
18S	Forward: AGTTCCAGCACATTTTGCGAG<br>Reverse: TCATCCTCCGTGAGTTCTCCA
Slc2a1	Forward: TGGCGGGAGACGCATAGTTA<br>Reverse: AACTCCTCAATAACCTTCTGGGG
Pdk1	Forward: GGGCCAGGTGGACTTCTATG<br>Reverse: TGGATATACCAACTTTGCACCAG
Pdk3	Forward: TTCAATGCCAAAGCGCCAAA<br>Reverse: AAGTGGGACTCCACCACCTA
Pdk4	Forward: AAACCGTCCTTCCTTGACCC<br>Reverse: AAACCAGCCAAAGGGGCATT
Ldha	Forward: AACTTGGCGCTCTACTTGCT<br>Reverse: GGACTTTGAATCTTTTGAGACCTTG

Detailed Methods

ESC culture

Undifferentiated J1 mouse ESCs (mESCs) were cultured on mitomyocin C (Sigma-Aldrich) treated mouse embryonic fibroblasts (MEFs) feeder layer in Dulbecco's modified Eagle's medium (DMEM) (Gibco) supplemented with 15% fetal bovine serum (FBS) (Invitrogen), glutamax (Invitrogen), non-essential amino acids (Invitrogen), penicillin/streptomycin (Invitrogen), 55mM 2-mercaptoethanol (Invitrogen) and 1000 U/ml leukemia inhibitory factor (LIF) (Millipore). Prior to endothelial differentiation, cells were passaged onto 0.1% gelation coated plates using the same medium. MEFs were maintained in DMEM containing 10% fetal bovine serum (FBS). Undifferentiated H1 human ESCs (hESCs) were cultured on Matrigel-coated plates with Essential 8 medium (Gibco).

Endothelial differentiation during hypoxia

To induce mESC differentiation towards the endothelial lineage, seven hundred cells/cm^2 of mESCs were seeded on collagen IV-coated plate in the absence of LIF and maintained in endothelial cell differentiation medium (ECDM) for 7 days either under normoxia (21% O_2) or in a hypoxic chamber (Biospherix) calibrated to maintain low oxygen tension (1% O_2, 5% CO_2, and balanced with N_2). ECDM medium contained 50% Iscove's Modified Dulbecco's Medium (Gibco), 50% Ham's F-12 medium (Gibco), supplemented with N2 (Gibco), B27 retinoic acid (Gibco), 0.05% lipid-rich bovine serum albumin (BSA) (Gibco), 50 ng/ml of ascorbic acid (Sigma) and 50 ng/ml of monothioglycerol (MTG) (Sigma), 50 ng/ml of human vascular endothelial growth factor (hVEGF), 10 ng/ml of human basic fibroblast growth factor (bFGF), and 2 ng/ml of human bone morphogenetic protein 4 (BMP4). This differentiation protocol generates functional endothelial cells which express endothelial transcription factors and endothelial-specific cell-surface markers VE-cadherin, form tubes *in vitro* and blood vessels *in vivo*(Toya et al., 2015).

To differentiate hESCs towards an endothelial lineage, ten thousand cells/cm^2 were seeded on matrigel-coated plate in Essential 8 medium and incubated for 24 hours (Day0) Next day (Day1), Essential 8 medium was replaced with human endothelial cell differentiation medium (50% Iscove's Modified Dulbecco's Medium (Gibco), 50% Ham's F-12 medium (Gibco), 1x Insulin-transferrin-selenium-X (Gibco), 1% Chemically defined lipid concentrate (Gibco), 5 mg/ml BSA (Sigma), 50 ng/ml ascorbic acid (Sigma) and 200μM 1-Thioglycerol (Sigma) supplemented with 25ng/ml Activin A (Stemgent), 10ng/ml BMP4 (R&D), 50ng/ml VEGF (PeproTech) and CHIR 1.5μM (Stemgent) and incubated for 3 days. At Day3, Day5 and Day7, medium was replaced with human serum free differentiation medium supplemented with 50ng/ml VEGF (PeproTech) and 10μM SB431542 (Stemgent). Differentiated cells at Day8 were collected for analysis by flow cytometry.

Gene deletion studies with CRISPR/Cas9

Mouse ESCs were co-transfected with plasmids containing Cas9, Hif1α-specific gRNA (Santa Cruz) and GFP as well as homology-directed repaired (HDR) plasmids (Santa Cruz) containing homology arms corresponding to the cut sites generated by Hif1α-specific gRNA. HDR plasmids inserted a puromycin resistance gene to the sites targeted by the Hif1α-specific gRNA. Two days after transfection, GFP positive mESC were sorted and plated on feeder cells for 5 days. Resultant single mESC colonies were then picked and expanded under puromycin selection (1.8μg/ml). Hif1α protein expression of each clone was then assessed in hypoxia-exposed mESC.

A *Etv2*$^{-/-}$ and *Notch1*$^{-/-}$ mESC line was generated by co-transfecting px330 plasmid(Cong et al., 2013) containing gRNA specific to Etv2 and Notch1 and puromycin resistance containing plasmid. Following 7 days of puromycin selection single mESC colonies were then picked and expanded. Each *Etv2*$^{-/-}$ and *Notch1*$^{-/-}$ clone was subjected to EC differentiation and mRNA expression of was measured on Day3 for *Etv2* and Day 7 for *Notch1* to validate the successful knockout.

Generation of *Pecam1*-GFP mESC reporter line

To track the fate of the injected cells *in vivo*, a mESC reporter line was generated using CRISPR/Cas9. Green fluorescence protein (GFP) was incorporated within the exon one of *Pecam1* gene; i.e. GFP expression is driven by endogenous *Pecam1* promoter. The sequence of gRNA used for targeting mouse *Pecam1* locus was 5' CAGCTGAGGTGGGCCTCAGT 3'

Chromatin immuno-precipitation (ChIP) assay

ChIP was performed using a High-Sensitivity ChIP Kit (Abcam) per the manufacturer's instructions. mESC at day3 of endothelial differentiation were fixed with 1.5% formaldehyde (Sigma) for 10 minutes. Glycine was then added to a final concentration of 125mM to quench the excess formaldehyde. Cells were then collected and washed twice with PBS containing protease inhibitors. Washed cells were lysed with lysis buffer at cell density of one million cells/200µl. Chromatin pellets were then resuspended in ChIP buffer and subjected to chromatin shearing (Covaris S2) for 40s at 7°C to reach 500bp fragments. Two-micrograms of chromatin per each sample were used to bind in one Hif1α antibody-coated well. Wash and elution steps were followed as described in the manufacturer's manual. Four microliter of resultant DNA were used for quantification by quantitative real-time PCR using primers (Forward 5' AAGGGACCATTCCAGGGACC 3' and Reverse 5' GGGTCGCAGCCTGGGTCAA 3') detecting HRE-containing Etv2 promoter region.

Real-time PCR

Total RNA was prepared using QIAshredder and RNeasy mini kit (Qiagen, Inc.) per manufacturer's instructions. RNA (1µg) was converted into cDNA using High-Capacity cDNA Reverse Transcription Kit (ABI). QPCR was performed by fast SYBR green PCR master mix (ABI) with specific primers (Table S1). Samples were run on a ViiA7 QPCR machine (Life Technologies).

FACS analysis

Endothelial cells were quantified using FACS analysis. VE-cadherin and PECAM1 were used as positive markers to determine the differentiation of mESCs into ECs. Differentiated cells differentiation of mESCs were harvested with 0.05% trypsin-EDTA (Invitrogen) and incubated with anti-VE-cadherin at 1:200 (R&D Systems #AF1002) for one hour. Cells were then washed twice with 0.2%BSA in PBS buffer. Donkey anti-goat Alexa-fluor 488 (1:500) (Molecular Probe #A-11055) and APC-conjugated anti-PECAM1 (BD Biosciences#565509) were then incubated with the cells for 1hour. Normal Goat IgG (R&D Systems #AB108C) and APC-Rat IgG2a Kappa(BD Biosciences#564982) were used as negative controls for FACS gating. Labeled cells were analyzed by Cyan ADP flow cytometer (Beckman Coulter).

Cell Cycle Analysis

BrdU labeling and flow cytometric quantification was used to determine the effect of hypoxia on the proliferation of mESCs undergoing endothelial differentiation. mESCs were collected on day 5 of endothelial differentiation and labeled using a BD Pharmingen APC BrdU Flow Kit per manufacturer's instructions. Flk1 (eBioscience #12-5821-82), a marker for vascular progenitors(Yamashita et al., 2000), was used to gate for early endothelial cells.

Immunofluorescence

mESCs were differentiated on collagen IV-coated coverslips. At Days 3, 4 and 5 of differentiation, cells were fixed with 4% paraformaldehyde (PFA) for 30 minutes. Excess PFA was quenched with glycine for 10 minutes. Cells were then blocked with 10% donkey serum in 0.5%BSA/PBS buffer. The primary antibody against VE-cadherin (Santa Cruz Biotechnology #sc-9989) and Etv2 (Abcam #181847) was incubated with the cells for 1 hour. Following washing, donkey anti-goat Alexa-546 (Molecular Probes #A-11056) and donkey anti-rabbit Alexa-488 (Molecular Probes #A-11055) were added and incubated for 1 hour. Coverslips were mounted using ProLong® Gold Antifade Mountant containing DAPI (Molecular Probe #P36931). Images were acquired using a confocal microscope (Carl Zeiss LSM710).

Western blotting

mESCs or differentiated cells were harvested on Day 0 (6-hour incubation after seeding), 3, 5, and 7 and lysed in RIPA lysis buffer (Santa Cruz) containing protease and phosphatase inhibitors (Santa Cruz). Protein concentrations were measured using Pierce™ BCA Protein Assay Kit (Thermo Scientific). Denatured protein (30µg) was resolved in SDS-PAGE and immunoblotted on PVDF membrane. Protein of interests were detected using primary antibodies against HIF1α (Caymen Chemicals #10006421), HIF2α (Novus Biologicals #NB100-122), VE-cadherin

(Santa Cruz #sc-9989), NOTCH1 (Cell Signaling#3603), cleaved NOTCH1 (Cell Signaling#4147), EPHRINB2 (R&D system #AF496), COUPTFII (Cell Signaling #6434) and β-actin (Abcam #ab8227).

Mouse ischemia models

The *Pecam1*-driven GFP reporter mESC line was used to differentiate endothelial cell under hypoxic and normoxic conditions as described. At day 7 of endothelial differentiation, GFP+ cells were sorted by FACS. For the mouse myocardial infarction (MI) model, ten to twelve-week old C57/BL6 mice were subjected to left thoracotomy after anesthetization with 1.5-3% isoflurane inhalation and Etomidate i.p. injection. Surgical anesthesia was maintained using 1% isoflurane delivered through mechanical ventilation. Ligation of the left main coronary artery was performed to induce infarction(Mavrommatis et al., 2013). Immediately after ligation, $0.5x10^6$ hypoxia-derived GFP^+ cells, $0.5x10^6$ normoxia-derived GFP^+ cells, or $0.5x10^6$ primary mouse adult endothelial cells (negative control) in or Matrigel alone (no cell control) were introduced by direct intramuscular injections into the peri-infarcted regions of the myocardium. For the mouse hind limb ischemia model, male athymic nude mice obtained from Jackson Laboratory at 8-9 weeks of age were subjected to unilateral hindlimb surgery under anesthesia with intraperitoneal administration of ketamine (87 mg/kg) and xylazine (13 mg/kg). We performed ligation and segmental resection of left femoral vessels followed by physiological and histological analysis as previously described with minor modifications(Urao et al., 2008). Briefly, the left femoral artery was exposed, ligated both proximally and distally using 6-0 silk sutures and the vessels between the ligatures was excised without damaging the femoral nerve. All arterial branches between the ligations were obliterated using an electrical coagulator (Fine Scientific Tools). ESC-derived ECs or mature ECs ($1x10^6$ cells/100ul) were injected into three different spots in gastrocnemius muscles right after ischemic surgery. Skin closure was done using 6-0 nylon sutures. The Animal Care and Use Committee of the University of Illinois at Chicago approved the study protocols. We measured ischemic (left)/nonischemic (right) limb blood flow ratio using a laser Doppler blood flow (LDBF) analyzer (PeriScan PIM 3 System; Perimed) as we described(Urao et al., 2008). Mice were anesthetized and placed on a heating plate at 37°C for 10 minutes to minimize temperature variation. Before and after surgery, LDBF analysis was performed in the plantar sole. Blood flow was displayed as changes in the laser frequency, represented by different color pixels, and mean LDBF values were expressed as the ratio of ischemic to non-ischemic LDBF.

Immunohistochemistry and engraftment analysis

Infarcted heart and gastrocnemius (GC) muscles of ischemic hindlimbs were collected at Day21 and Day 28 post-surgery, respectively. Tissues were fixed in 4% paraformaldehyde for 24 hours and then were embedded in paraffin. Tissue slices with 5μm thickness were then obtained. To perform immunohistochemistry, slides were rehydrated and antigen retrieval was done with citrate buffer for 5 minutes. They were then blocked with 0.3% hydrogen peroxide (Sigma) for 20 minutes, followed by three 10 minute washes with PBS containing 0.05% Tween20 (Sigma). Tissue slices were blocked with 10% donkey serum in 2% BSA/PBS for 1 hour. GFP (Abcam#6556), PECAM1 (Abcam#28364), Ki67(Abcam#15580) antibody was then incubated overnight at 4°C. After three 10 minute washes, donkey anti-rabbit was incubated for 1 hour. The slides were developed with DAB (Invitrogen) for 5 minutes and counterstained with hematoxylin (Sigma), followed by rinsing in running tap water for 10 minutes. Finally, the slides were dehydrated and mounted for imaging using Olympus BX51 microscope.

GFP, PECAM1, and Ki67 were stained on consecutive slices from infarcted hearts to visualize the location of GFP positive cells. Number of GFP cells in infarcted heart was counted from 3 animals and images of 6 fields were taken from each heart. Cell proliferation of transplanted cells were analyzed by quantifying the number of GFP^+ and Ki67+ cells from 3 animals in 8 fields of view at 20X magnification on Day21. To quantify the vessels density, number of $PECAM1^+$ cells in GC muscles was counted from 3 animals and 8 fields were selected from each.

Statistical analysis

All statistical analyses were performed with GraphPad Prism (San Diego). Results are expressed as mean ± standard deviation (SD). The differences between groups were analyzed using ANOVA and Student *t*-test. *p* values less than 0.05 was considered as statistically significant.

Supplemental References

Cong, L., Ran, F.A., Cox, D., Lin, S., Barretto, R., Hsu, P.D., Wu, X., Jiang, W., and Marraffini, L.A. (2013). Multiplex Genome Engineering Using CRISPR/VCas Systems. Science (80-.). *339*, 819–823.

Mavrommatis, E., Shioura, K.M., Los, T., and Goldspink, P.H. (2013). The E-domain region of mechano-growth factor inhibits cellular apoptosis and preserves cardiac function during myocardial infarction. Mol. Cell. Biochem. *381*, 69–83.

Toya, S.P., Wary, K.K., Mittal, M., Li, F., Toth, P.T., Park, C., Rehman, J., and Malik, A.B. (2015). Integrin a6b1 Expressed in ESCs Instructs the Differentiation to Endothelial Cells. Stem Cells *33*, 1719–1729.

Urao, N., Inomata, H., Razvi, M., Kim, H.W., Wary, K., McKinney, R., Fukai, T., and Ushio-Fukai, M. (2008). Role of nox2-based NADPH 0xidase in bone marrow and progenitor cell function involved in neovascularization induced by hindlimb ischemia. Circ. Res. *103*, 212–220.

Yamashita, J., Itoh, H., Hirashima, M., Ogawa, M., Nishikawa, S., Yurugi, T., Naito, M., Nakao, K., and Nishikawa, S. (2000). Flk1-positive cells derived from embryonic stem cells serve as vascular progenitors. Nature *408*, 92–96.
